# Drinking Water Quality and Human Health: An Editorial

**DOI:** 10.3390/ijerph16040631

**Published:** 2019-02-21

**Authors:** Patrick Levallois, Cristina M. Villanueva

**Affiliations:** 1 Direction de la santé environnementale et de la toxicologie, Institut national de la santé publique du Québec, QC G1V 5B3, Canada; 2 Département de médecine sociale et préventive, Faculté de médecine, Université Laval, Québec, QC G1V 0A6, Canada; 3 ISGlobal, 08003 Barcelona, Spain; cristina.villanueva@isglobal.org; 4 Universitat Pompeu Fabra (UPF), 08002 Barcelona, Spain; 5 Consortium for Biomedical Research in Epidemiology and Public Health (CIBERESP), Carlos III Institute of Health, 28029 Madrid, Spain; 6 IMIM (Hospital del Mar Medical Research Institute), 08003 Barcelona, Spain

Drinking water quality is paramount for public health. Despite improvements in recent decades, access to good quality drinking water remains a critical issue. The World Health Organization estimates that almost 10% of the population in the world do not have access to improved drinking water sources [1], and one of the United Nations Sustainable Development Goals is to ensure universal access to water and sanitation by 2030 [2]. Among other diseases, waterborne infections cause diarrhea, which kills nearly one million people every year. Most are children under the age of five [1]. At the same time, chemical pollution is an ongoing concern, particularly in industrialized countries and increasingly in low and medium income countries (LMICs). Exposure to chemicals in drinking water may lead to a range of chronic diseases (e.g., cancer and cardiovascular disease), adverse reproductive outcomes and effects on children’s health (e.g., neurodevelopment), among other health effects [3].

Although drinking water quality is regulated and monitored in many countries, increasing knowledge leads to the need for reviewing standards and guidelines on a nearly permanent basis, both for regulated and newly identified contaminants. Drinking water standards are mostly based on animal toxicity data, and more robust epidemiologic studies with an accurate exposure assessment are rare. The current risk assessment paradigm dealing mostly with one-by-one chemicals dismisses potential synergisms or interactions from exposures to mixtures of contaminants, particularly at the low-exposure range. Thus, evidence is needed on exposure and health effects of mixtures of contaminants in drinking water [4].

In a special issue on “Drinking Water Quality and Human Health” *IJERPH* [5], 20 papers were recently published on different topics related to drinking water. Eight papers were on microbiological contamination, 11 papers on chemical contamination, and one on radioactivity. Five of the eight papers were on microbiology and the one on radioactivity concerned developing countries, but none on chemical quality. In fact, all the papers on chemical contamination were from industrialized countries, illustrating that microbial quality is still the priority in LMICs. However, chemical pollution from a diversity of sources may also affect these settings and research will be necessary in the future.

Concerning microbiological contamination, one paper deals with the quality of well water in Maryland, USA [6], and it confirms the frequent contamination by fecal indicators and recommends continuous monitoring of such unregulated water. Another paper did a review of Vibrio pathogens, which are an ongoing concern in rural sub-Saharan Africa [7]. Two papers focus on the importance of global primary prevention. One investigated the effectiveness of Water Safety Plans (WSP) implemented in 12 countries of the Asia-Pacific region [8]. The other evaluated the lack of intervention to improve Water, Sanitation and Hygiene (WASH) in Nigerian communities and its effect on the frequency of common childhood diseases (mainly diarrhea) in children [9]. The efficacies of two types of intervention were also presented. One was a cost-effective household treatment in a village in South Africa [10], the other a community intervention in mid-western Nepal [11]. Finally, two epidemiological studies were conducted in industrialized countries. A time-series study evaluated the association between general indicators of drinking water quality (mainly turbidity) and the occurrence of gastroenteritis in 17 urban sites in the USA and Europe. [12] The other evaluated the performance of an algorithm to predict the occurrence of waterborne disease outbreaks in France [13].

On the eleven papers on chemical contamination, three focused on the descriptive characteristics of the contamination: one on nitrite seasonality in Finland [14], the second on geogenic cation (Na, K, Mg, and Ca) stability in Denmark [15] and the third on historical variation of THM concentrations in french water networks [16]. Another paper focused on fluoride exposure assessments using biomonitoring data in the Canadian population [17]. The other papers targeted the health effects associated with drinking water contamination. An extensive up-to-date review was provided regarding the health effects of nitrate [18]. A more limited review was on heterogeneity in studies on cancer and disinfection by-products [19]. A thorough epidemiological study on adverse birth outcomes and atrazine exposure in Ohio found a small link with lower birth weight [20]. Another more geographical study, found a link between some characteristics of drinking water in Taiwan and chronic kidney diseases [21]. Finally, the other papers discuss the methods of deriving drinking water standards. One focuses on manganese in Quebec, Canada [22], another on the screening values for pharmaceuticals in drinking water, in Minnesota, USA [23]. The latter developed the methodology used in Minnesota to derive guidelines—taking the enhanced exposure of young babies to water chemicals into particular consideration [24]. Finally, the paper on radioactivity presented a description of Polonium 210 water contamination in Malaysia [25].

In conclusion, despite several constraints (e.g., time schedule, fees, etc.), co-editors were satisfied to gather 20 papers by worldwide teams on such important topics. Our small experience demonstrates the variety and importance of microbiological and chemical contamination of drinking water and their possible health effects.
